# Hook plate fixation for acute acromioclavicular dislocations without coracoclavicular ligament reconstruction: a functional outcome study in military personnel

**DOI:** 10.1007/s11751-015-0228-0

**Published:** 2015-07-28

**Authors:** Narinder Kumar, Vyom Sharma

**Affiliations:** Military Hospital, Kirkee, Pune, Maharashtra 411020 India

**Keywords:** Acromioclavicular dislocation, Hook plate, Military personnel

## Abstract

The aim of our study was to evaluate the shoulder function after clavicular hook plate fixation of acute acromioclavicular dislocations (Rockwood type III) in a population group consisting exclusively of high-demand military personnel. This prospective study was carried out at a tertiary care military orthopaedic centre during 2012–2013 using clavicular hook plate for management of acromioclavicular injuries without coracoclavicular ligament reconstruction in 33 patients. All patients underwent routine implant removal after 16 weeks. The functional outcome was assessed at 3, 6 and 12 months after hook plate removal and 2 years from the initial surgery using the Constant Murley and UCLA Scores. All the patients were male serving soldiers and had sustained acromioclavicular joint dislocation (Rockwood type III). Mean age of the patient group was 34.24 years (21–55 years). The mean follow-up period in this study was 23.5 months (20–26 months) after hook plate fixation and an average of 19.9 months (17–22 months) after hook plate removal. The average Constant Score at 3 months after hook plate removal was 60.3 as compared to 83.7 and 90.3 at 6 months and 1 year, respectively, and an average of 91.8 at the last follow-up that was approximately 2 years after initial surgery which was statistically significant (*p* value <0.05). The UCLA Score was an average of 15.27, 25.9 and 30.1 at 3, 6 months and 1 year, respectively, after removal of hook plate which improved further an average of 32.3 at the last follow-up, which was also statistically significant (*p* value <0.05). Clavicular hook plate fixation without coracoclavicular ligament reconstruction is a good option for acute acromioclavicular dislocations producing excellent medium-term functional results in high-demand soldiers.

## Introduction

Acromioclavicular joint injuries are a common entity with an ever-evolving approach towards management of these injuries from the days of Hippocrates [1] and Galen [2]. The quantum of these injuries is on the rise constituting approximately 9–12 % of all shoulder injuries following fall on an outstretched hand [3–6]. The commonly used and validated classification proposed by Rockwood divides these injuries into six types [7]. Though there is general consensus about conservative management for Rockwood type I and II injuries and surgical treatment for Rockwood type IV, V and VI injuries, the most suitable treatment for Rockwood type III injuries remains controversial [8–11].

Different approaches have been described for management of these injuries ranging from conservative management with bandages and slings to multiple surgical options including fixation of the acromioclavicular joint with pins, tension band wiring, the modified Weaver–Dunn procedure, fixation with washer and screw, suspensory fixation devices and clavicular hook plate. All of these options have their own specific advantages and disadvantages, but no clearly superior option has been established as yet [12].

The clavicular hook plates are pre-contoured plates with varying sizes and depths as well as side to fit different anatomy. After reduction in the acromioclavicular joint, the hook is placed under the acromion process posteriorly and the screws are used to fix the plate to lateral clavicle maintaining the reduction. The manufacturers of the plate recommend routine removal of the plate after 3 months to avert the complications of subacromial impingement and acromial osteolysis. Clavicular hook plates have been demonstrated to be an effective implant option for surgical treatment of Rockwood type III acromioclavicular dislocation but concerns have been raised about acromial osteolysis, subacromial impingement and even possibly rotator cuff injuries [13–15].

In view of absence of any concrete evidence for an ideal implant for fixation of a Rockwood type III acromioclavicular joint dislocation and necessity of coracoclavicular ligament reconstruction, we undertook this prospective study to establish the efficacy of clavicular hook plate for fixation of acute type III injuries without coracoclavicular/acromioclavicular ligament reconstruction in soldiers involved in high-demand activities and athletics.

## Materials and methods

The study design was a prospective study at a tertiary care military orthopaedic centre during 2012–2013 for management of acromioclavicular injuries. All patients with Rockwood type III acromioclavicular injuries were included in the study after approval of the institutional ethical committee. Exclusion criteria included Rockwood type I, II, IV, V, VI injuries, open injuries, polytrauma, neurovascular injury and concomitant shoulder or upper limb trauma. No other management modalities, including conservative management, were employed.

**Fig. 1:**
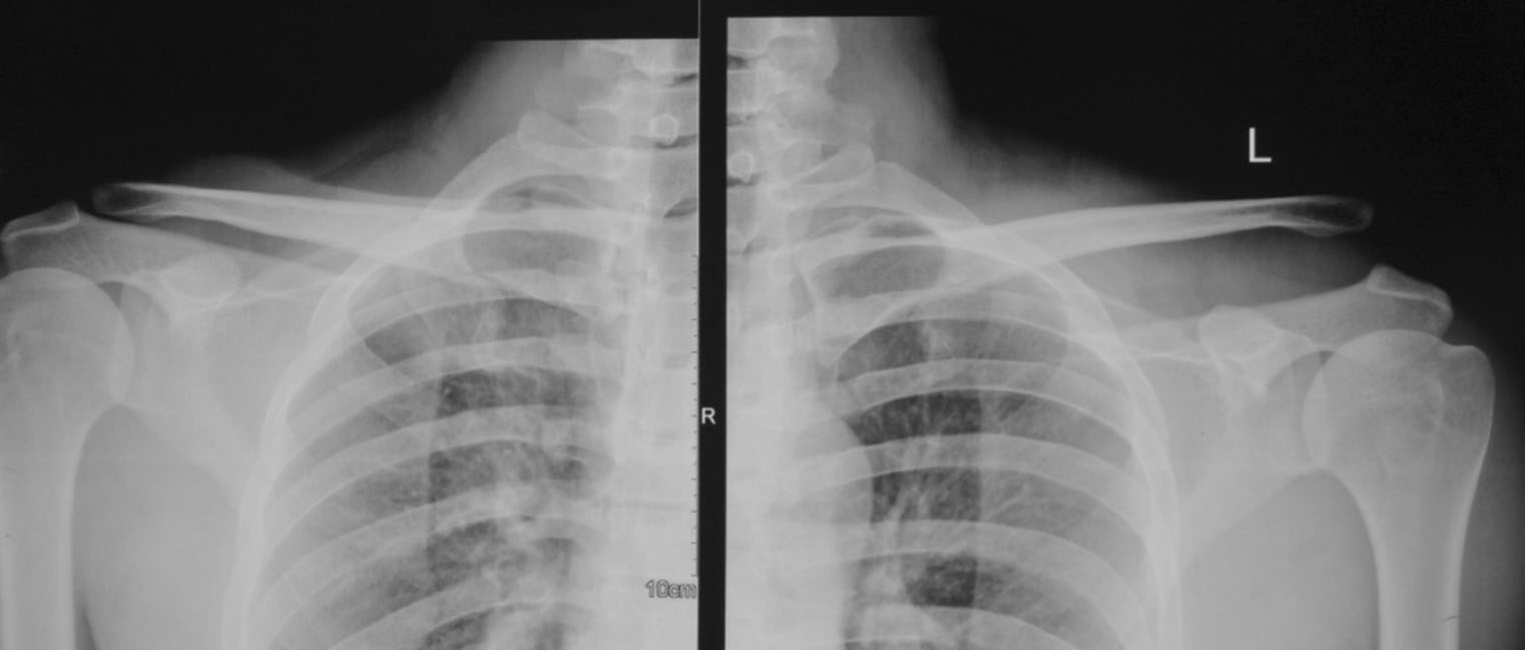
Pre-operative radiograph showing grade III acromioclavicular dislocation and an increased coracoclavicular distance

All the patients were subjected to radiographic analysis of an anteroposterior view of the shoulder and stress views which were accordingly classified by the attending surgeon [16] The radiographs were also assessed for coracoclavicular distance comparing in the injured versus noninjured shoulder (Fig. 1). Type III acromioclavicular injuries were treated surgically within 48 h of arrival at the centre with open reduction and fixation with clavicular hook plate (DePuy Synthes) in beach chair position under general anaesthesia. Surgery was delayed in some cases due to concomitant injuries or delay in referral of the patient to our centre. The surgical approach was a transverse incision over lateral third of clavicle. The acromioclavicular joint was exposed after assessing the torn acromioclavicular ligaments. The fixation of the acromioclavicular separation was done with titanium clavicular hook plate (4, 5 or 6 hole) in templated hook offset (12, 15 or 18 mm) without any supplemental ligamentous repair or reconstruction of coracoclavicular or acromioclavicular ligaments. Post-operatively, arm sling was used for 10 days to 2 weeks. Passive- and active-assisted shoulder range of motion (ROM) was commenced on second post-operative day as per pain tolerance. Active shoulder movements including abduction up to 90° were initiated 2 weeks post-operatively onwards.

All patients were taken up for removal of the hook plate after a mean period of 16 weeks (14–22 weeks) and subsequently enrolled in an institutional shoulder rehabilitation programme to regain shoulder range of motion including cuff-strengthening exercises. The patients were followed up for a minimum period of 24 months after hook plate fixation. The patients were subjected to radiographic assessment at 12 weeks, 6 months, 1 and 2 years which included the congruency of acromioclavicular joint and restoration of the coracoclavicular distance or any increase in the same at later follow-up examinations. The functional outcome was assessed using the Constant and Murley Score and UCLA Score with assessment at all follow-ups. The Constant Score and UCLA Score prior to and following hook plate removal were subjected to paired *t* test for statistical significance.

## Results

A total of 45 patients with acromioclavicular dislocations were managed at a tertiary military orthopaedic centre from Dec 2011 to Apr 2013. The study population comprised of soldiers who were diagnosed with Rockwood type III acromioclavicular dislocation. The sample size was eventually thirty-three soldiers after excluding Rockwood type I, II, IV, V, VI injuries, open injuries, polytrauma, neurovascular injury and concomitant shoulder or upper limb trauma (12 cases excluded). All the included patients were male. The mean age of the patients was 34.24 years (21–55 years) with 40 % in their thirties and 32 % in twenties. The common mechanism of injury was fall on shoulder or outstretched hand following sports injuries (60 %) and road traffic accident (28 %). All the patients had acute injuries (less than 2 weeks). Twenty-three patients (69 %) had injury in the nondominant arm. The average duration of surgical intervention from the day of injury was 9.06 days (4–15 days). All the patients in this study had Rockwood’s type III acromioclavicular dislocation. The operating surgeons varied from residents to consultants with experience ranging from 2 to 15 years. The mean duration of the procedure was 43 min (35–55 min). The average length of the incision was 84.2 mm (70–100 mm). The most commonly used hook plate was 5 holes in twenty-four (72 %) patients with 18 mm hook offset. There was no incidence of surgical site infection or any post-operative complications. The average hospital stay was 7.6 days after surgery (5–10 days) as all soldiers undergo supervised rehabilitation. The hospital stay was longer than usual due to peculiar nature of clientele (soldiers) which hails from all parts of the country. The hospital caters for extra beds required for convalescence till suitable arrangements can be made for convalescing soldier to travel home. The hospital stay includes stay in convalescence beds which would normally be at home in other facilities.

The patients were taken up for removal of the hook plate after an average period of 16 weeks (14–22 weeks) from the day of surgery with 48 % patients in 14- to 16-week period. There were three patients (9 %) who reported after the stipulated period of implant removal (12–14 weeks) at 20–22 weeks.

The mean follow-up period in this study was 23.5 months (20–26 months) after hook plate fixation and an average of 19.9 months (17–22 months) from the day of hook plate removal.

**Fig. 2:**
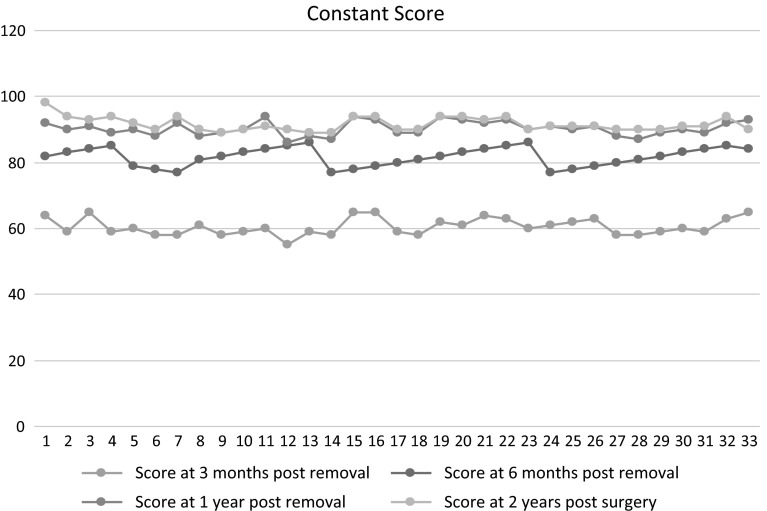
Constant Score at 3, 6 months and 1 year after hook plate removal and 2 years post hook plate fixation

**Fig. 3:**
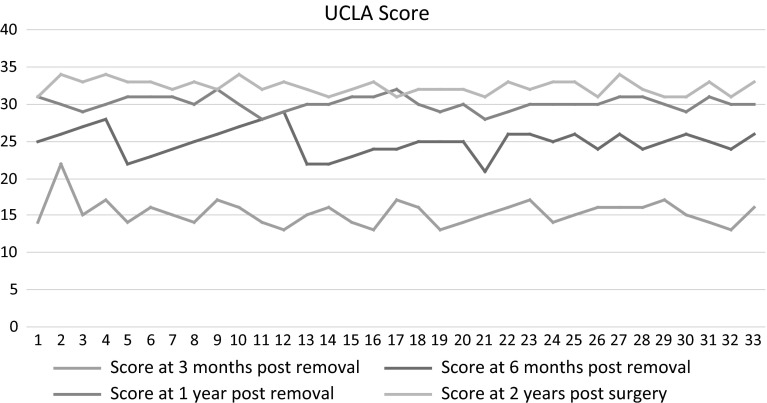
UCLA Score at 3, 6 months and 1 year after hook plate removal and 2 years post hook plate fixation

The functional outcome was assessed after hook plate removal at all follow-ups. The average Constant Score at 3 months after hook plate removal was 60.3 (95 % Confidence Interval between 58.7 and 61.9) as compared to 83.7 and 90.3 at 6 months and 1 year, respectively, and an average of 91.8 (95 % Confidence Interval between 88.5 and 93.05) at the last follow-up which was approximately 2 years after initial surgery. This was statistically significant (*p* value <0.05) as shown in Fig. 2. The UCLA Score was an average of 15.27 (95 % Confidence Interval between 14.6 and 15.8), 25.9 and 30.1 at 3, 6 months and 1 year, respectively, after removal of hook plate which was further an average of 32.3 (95 % Confidence Interval between 31.9 and 32.6) at the last follow-up, which was also statistically significant (*p* value <0.05) as shown in Fig. 3.

The significant functional limitations in the period prior to hook plate removal were mild-to-moderate pain in 15 (45 %) patients, restricted overhead abduction and terminal internal rotation in majority of patients. The functional evaluation at the last follow-up revealed that none of the patients had pain in the affected shoulder and had achieved full overhead abduction. All the patients had returned to pre-injury activity level including sports except one patient who felt moderate impairment in this regard though he had achieved full range of painless motion. None of the patients had any recurrence of instability after hook plate removal.

**Fig. 4:**
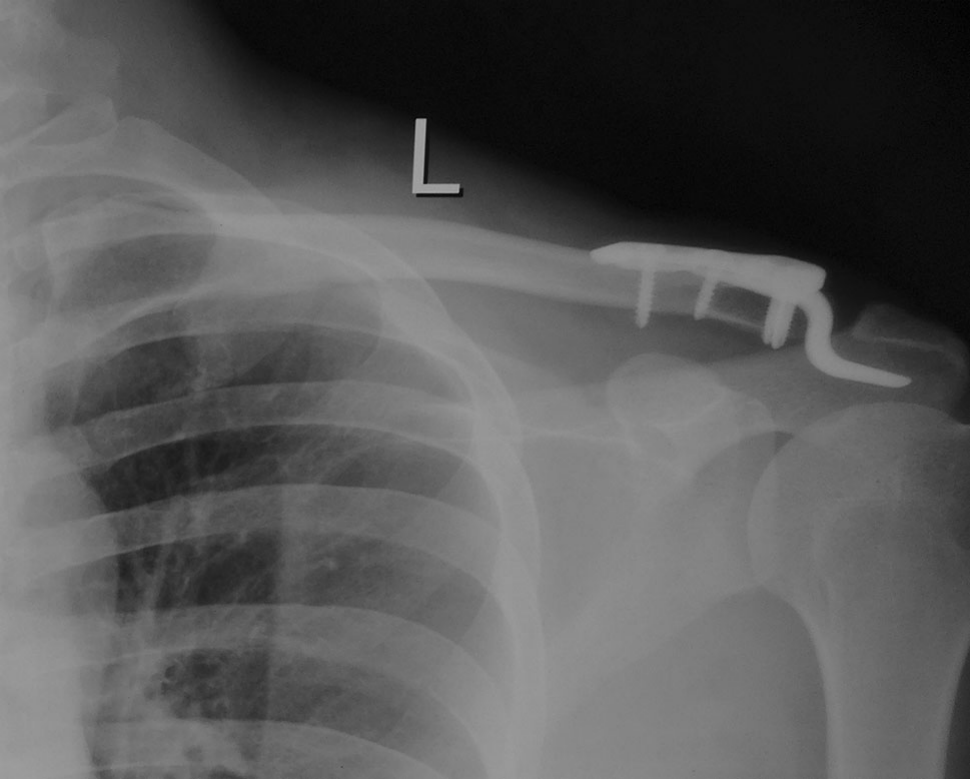
Post-operative radiograph at 12 weeks showing a congruent acromioclavicular joint and hook plate in situ

The radiological assessment at 12–14 weeks (prior to hook plate removal) by plain radiographs revealed congruent acromioclavicular joint and no evidence of osteolysis and comparable coracoclavicular distance (Fig. 4).

**Fig. 5:**
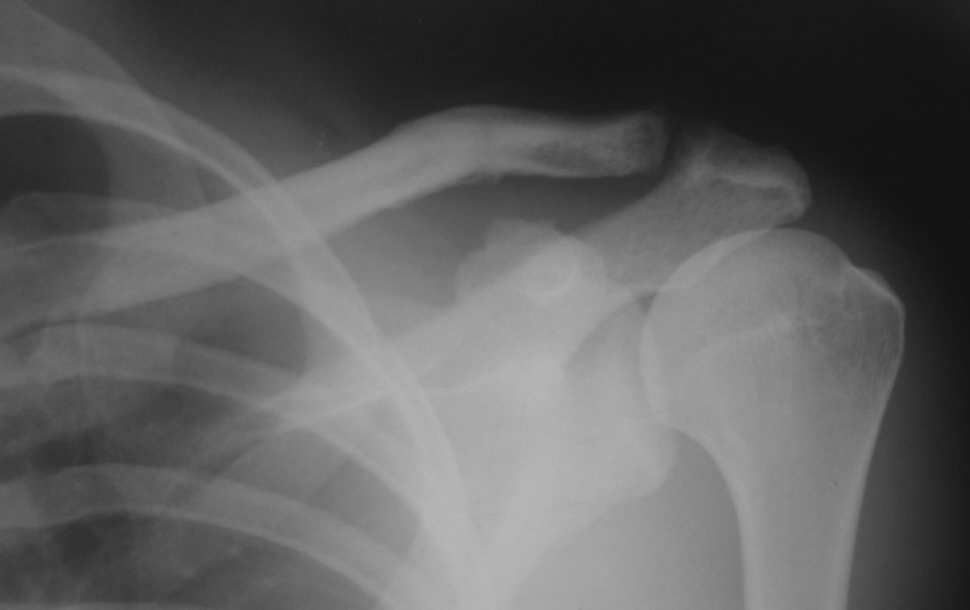
Stress radiograph at 2 years post hook plate fixation: Congruent acromioclavicular articulation and normal coracoclavicular distance with mild sclerosis

The follow-up radiographs immediately after hook plate removal revealed no subluxation or dislocation and a comparable coracoclavicular distance to the unaffected shoulder on stress radiograph. There was no evidence of osteolysis at the last follow-up post hook plate removal, and screw tracks had healed adequately. There was evidence of sclerosis in acromion and distal end clavicle in three cases, though the patients were completely asymptomatic with full functional recovery (Fig. 5).

## Discussion

There has been a shift in the management of acromioclavicular injuries with an ever-increasing consensus towards nonoperative treatment for Rockwood type I–II and surgical treatment for type IV–VI [17]. However, despite more than 150 surgical techniques being described for type III acromioclavicular joint dislocation, there is still no consensus on the ideal fixation method/device for fixation of a type III dislocated acromioclavicular joint [18]. The debate of nonoperative versus operative treatment for type III injuries remains undecided as studies have found advantages and disadvantages of both in young athletic population [19].

The probability of any surgical procedure and fixation device to maintain a congruent acromioclavicular joint and a good shoulder function is dependent on the fixation device which mimics the biomechanics of native acromioclavicular joint. The role of Kirschner wires and pins for fixation of acromioclavicular dislocation has definitely gone into disrepute due to complications like pin breakage and pin migration [20]. The results of coracoclavicular screw with or without ligament reconstruction has also shown variable results in small sample of patients [21]. The basis of using an anatomically contoured clavicular hook plate is the proximity of this device to mimic the amphiarthrotic nature of the acromioclavicular articulation. In view of this, we preferred to use the hook plate in our subset of patients who were primarily athletic and involved in strenuous physical activities as soldiers. Recent literature has reported excellent results using tightrope (arthrex), but these results were not available at the time of our study to consider as an option [17].

The demographic pattern of acromioclavicular injuries has depicted a steep trend towards males sustaining these injuries with 50 % or more in the age group of 20–39 years [22]. Our study population reflected the same with 72 % of the patients belonging to this age group. The role of sport-induced factors in these injuries has been well established, and a large number of patients (60 %) in our study sustained injury during basketball, wrestling, cycling or even fall from a vertical rope in our study population [23].

The most significant disadvantages of conservative management of an acromioclavicular injury are an impaired shoulder function, pain, cosmetic deformity and effect on performance of athletes involved in upper limb activities. All the earlier fixation methods led to an extremely rigid fixation which impaired the rotational movement between clavicle and scapula [24]. This aspect is taken care of by an implant-like clavicular hook plate which forms leverage between proximal ends of plate fixed to distal clavicle; hook penetrates the undersurface of acromion and maintains the amphiarthrotic acromioclavicular articulation [25].

The functional outcome of shoulder following removal of the hook plate improved significantly during subsequent follow-ups. The Constant Score after 12 weeks post hook plate removal hook was on an average 60, primarily due to improving but painful shoulder motion and moderate pain. In this study, we did not allow the patients to attempt overhead abduction beyond 90 degrees, while hook plate was in situ to avoid inadvertent damage to acromion and subsequent acromial osteolysis and subacromial bursitis which has been reported in the earlier studies on hook plate fixation [26, 27]. There was a significant improvement in the Constant Score at 18 months post hook plate removal in all the patients. There were 19 patients (57 %) with Constant Score above 90 at the last follow-up. All the patients had significant improvement in overhead abduction (beyond 120°) and returned to active sports such as basketball, handball and kabaddi. The functional outcome was similarly excellent as seen in UCLA Scores at the last follow-up at around 2 years post-surgery. The ultimate goal of surgical intervention in this set of injuries was to facilitate return to their pre-injury level of active sports which was achieved in all the patients which is comparable to the results of earlier studies where hook plate has been used [26]. The major disadvantages of hook plate cited in earlier series have been repeat surgery, persistent shoulder pain, incomplete shoulder function, acromial osteolysis and acromioclavicular subluxation [27]. In our study, we had no surgical site infection, and in the early and midterm follow-up, there was no incidence of osteolysis, subluxation of acromioclavicular joint after hook plate removal. There was no requirement of any repeat surgical intervention other than the removal of hook plate itself. The concern of subacromial impingement was pertinent till the hook plate was in situ. However, after plate removal, there was no clinicoradiological evidence of the same. The radiological assessment of a sound acromioclavicular joint can be done with stress radiographs and measurement of the coracoclavicular distance comparing with the contralateral side or an absolute value which should be 11–13 mm generally [28].

The hook plate in our experience is an excellent device to obtain a congruent acromioclavicular joint due to its unique biomechanical characteristics and stiffness which are most similar to a physiologic acromioclavicular articulation [25].

The debate on surgery versus conservative treatment in type III injuries is not applicable to young athletic individuals, like soldiers in our study, in view of their high functional requirements which are met better with surgical stabilisation. An extension of the same debate is whether surgery restores the strength of ligaments. This has been proven by the excellent functional outcome in all the patients in our study with all the patients returning to their pre-injury athletic performance [29, 30]. The hook plate works quite well as an “internal splint” that keeps the acromioclavicular joint reduced during the time necessary for biological healing of the ligaments. In addition, the accuracy of joint reduction can be clearly visualised per-operatively. Though quite a few arthroscopic procedures have been described recently using suspensory fixation devices, an extremely high level of accuracy is required in terms of placement of tunnels. Faulty placement of tunnels may lead to fractures of coracoid or clavicle [31, 32]. Though these techniques are appealing in view of cosmesis and ability to treat concomitant shoulder injuries, the technique remains restricted to experienced shoulder arthroscopists. Good to excellent results have also been reported with open techniques using suspensory devices, e.g. tightrope [17]. In comparison, the results of hook plate fixation have been consistent in our study despite variable experience of the operating surgeons as the surgical technique is simple and easily reproducible.

The major drawback of using a hook plate is requirement of another surgery for removal of implant. Though there were no complications in our study, the hook plates can cause disturbances over the subacromial bursa, supraspinatus tendinitis, disturbances over the plate end and acromial osteolysis, if retained for long time. We were able to avoid these complications by timely removal of the implant. The limitation of our study is a relatively small sample size (thirty-three) and absence of a control group. A major advantage of our study was that the entire population group of soldiers was homogenous with similar functional requirements.

## Conclusion

It can be concluded that precontoured clavicular hook plate is a good implant option to be considered for fixation of type III acromioclavicular dislocations without requiring any additional ligamentous procedures. The recommendation to apply this conclusion across all types of acromioclavicular dislocations would not be absolutely pertinent as this study primarily dealt with type III injuries. Young active athletic patients like soldiers with such injuries would definitely benefit with an early reduction and fixation with hook plate followed by its timely removal.
